# A New Approach for Scatter Removal and Attenuation Compensation from SPECT/CT Images

**Published:** 2013-11

**Authors:** Shabnam Oloomi, Hadi Noori Eskandari, Seyed Rasoul Zakavi, Peter Knoll, Faraz Kalantari, Mohsen Hajizadeh Saffar

**Affiliations:** 1 Department of Medical Physics, Mashhad University of Medical Science, Mashhad, Iran; 2 Department of Applied Mathematics, School of Mathematical Sciences, Ferdowsi University of Mashhad, Mashhad, Iran; 3 Nuclear Medicine Research Center, Imam Reza Hospital, Faculty of Medicine, Mashhad University of Medical Sciences, Mashhad, Iran; 4 Department of Nuclear Medicine Wilhelminenspital Vienna, Austria; 5 Research Center for Nuclear Medicine, Tehran University of Medical Sciences, Tehran, Iran

**Keywords:** Attenuation correction, MLEM, Scatter correction, SPECT

## Abstract

***Objective(s):*** In SPECT, the sinogram contains scatter and lack of attenuated counts that degrade the reconstructed image quality and quantity. Many techniques for attenuation and scatter correction have been proposed. An acceptable method of correction is to incorporate effects into an iterative statistical reconstruction. Here, we propose new Maximum Likelihood Expectation Maximization (MLEM) formula to correct scattering and attenuating photons during reconstruction.

***Materials and Methods:*** In this work, scatters are estimated through Klein-Nishina formula in all iterations and CT images are used for accurate attenuation correction. Reconstructed images resulted from different MLEM reconstruction formula have been compared considering profile agreement, contrast, mean square error, signal-to-noise ratio, contrast-to-noise ratio and computational time.

***Results:*** The proposed formula has a good profile agreement, increased contrast, signal-to-noise (SNR) & contrast-to-noise ratio (CNR), computational time and decreased mean square error (MSE) compared with uncorrected images and/or images from conventional formula.

***Conclusion:*** In conclusion, by applying the proposed formula we were able to correct attenuation and scatter via MLEM and improve the image quality, which is a necessary step for both qualitative and quantitative SPECT images.

## Introduction

Single photon emission computed tomography (SPECT) is an imaging modality used to visualize the biological uptake and distribution of an applied radiopharmaceutical. The physical effects of attenuated and scattered photons have to be taken into account to improve the image quality. For non-uniform attenuators (for example in the human thorax), the generation of a patient attenuation map is necessary. Several attenuation correction methods have been reported and are used in clinical studies. Transmission computed tomography with an external gamma-ray source has been widely used in nuclear medicine for cardiac SPECT studies but is limited only to ^99m^Tc or ^201^Tl studies. The images obtained by this method can be used only for attenuation correction but not for anatomical orientation. In recent years, multi-modality imaging using techniques from two different modalities (PET or SPECT and x-ray CT) was developed. Attenuation coefficient maps generated from X-ray CT images have several advantages such as shorter acquisition time and improved image quality obtained by even low-dose CT scanning protocols which also enables the anatomic orientation. 

Over the last two decades, intensive efforts have been made to compensate for the scatter effect in SPECT in order to improve the quantitative and qualitative accuracy of the reconstructed images (1,2) . A class of widely used scatter compensation methods is based on the estimation of the scatter component in the photopeak projection data and subsequent subtraction or deconvolution of the scatter contribution from the measured projection data. Scatter compensation methods in this class are fast and simple, but increase the noise in the reconstructed images. Another class which is a promising approach for scatter compensation, consists of reconstruction-based scatter compensation methods (RBSC) (3) resulting in images with both less bias and reduced variance as compared with subtraction-based scatter compensation methods (4-6). 

RBSC methods are based on modeling the scattered photons in projection–back projection processes. Several techniques have been developed for calculating the scatter, one of them which was used in this study is based upon the integration of the Klein–Nishina formula in non-uniform media (3, 7, 8). 

In this work, we introduce new approach for both attenuation and scatter correction during reconstruction using the MLEM approach. To obtain a proposed iterative formula for reconstruction of the SPECT images, we utilize the necessary conditions to optimally maximize the likelihood function. Then, its importance is evaluated in inhomogeneous media of digital and experimental phantoms. Finally, the contrast, mean square error (MSE), signal-to-noise ratio (SNR), contrast-to-noise ratio (CNR) and computational time of the newly developed algorithm were compared with the conventional MLEM ones.

## Methods and Materials

***Proposed MLEM iterative formula***

The projections acquired in different angles around the object of interest can be used to reconstruct trans-axial slice images through analytical or iterative methods containing algebraic and statistical methods. Most commonly used iterative reconstruction method is maximum likelihood expectation maximization (MLEM) or ordered subset expectation maximization (OSEM), a faster implementation of MLEM algorithm (9-11). In this section, a proposed MLEM iterative formula for reconstruction of the SPECT image is described. Considering the following assumptions:

*n* for Number of detection bin, equal to detector pixels × projection number

*m* for Number of image pixels, *K* for number of iterations

*f*_i _*j=1,2,…,m* are the pixel values of the image, proportional to the number of radionuclide activity in pixel k 

*g*_i_ *i=1,2,…,m* are the sinogram measured data from i^th^ detection bin in gamma camera

*a*_ij_ *i=1,2,...,n* and *j =1,2,...,m* are the elements of the system matrix or detection probability of emitted photons from *aij i=1,2,…,n* pixel *j* of the subject to be detected in i^th^ detection bin in gamma camera.

$a_{\mathrm{ij}}\left(\mu \right)=a_{\mathrm{ij}}e^{-\sum k\in \mathrm{jrik\mu k}}$ are the attenuated system matrix elements, 

*r*_ik_ and *µ*_k _for *i=1,2,...,n* and *kԑj=1,2,...m* are the length and attenuation coefficient of pixels of number k, which are along the direction of pixel j to detection bin i

SC_i_ *i=1,2,…,n* are the appeared scatter photons in i^th^ detector of gamma camera. 

As g_i_ contains primary and scattered photons, so in each detector we have:

Equation 1 $$E\left(g_{i}\right)={\left(\mathrm{AF}\right)}_{i}+{\mathrm{SC}}_{i}$$

Where E(g_i_) is the mathematical expectancy of Poisson variable g_i_. The variable g_i_ have a possibility function such as: 

Equation 2 $$P\left(g_{i},{\mathrm{SC}}_{i}+\sum_{k=1}^{m}a_{\mathrm{ik}}\left(\mu \right)f_{k}\right)=\frac{{\left({\mathrm{SC}}_{i}+\sum_{k\in j=1}^{m}a_{\mathrm{ik}}\left(\mu \right)\mathrm{fk}\right)}^{g_{i}}}{g_{i}}\exp(-{\mathrm{SC}}_{i}-\sum_{k\in j=1}^{m}a_{\mathrm{ik}}\left(\mu \right)\mathrm{fk}$$

Here, we estimate the values of for *k=1,2,...,m* using the logarithm of maximum likelihood (LML) function, that is:

Equation 3 $$L\left(f\right)=\sum_{i=1}^{n}\left[g_{i}\mathrm{Ln}\left({\mathrm{SC}}_{i}+\sum_{k\in j=1}^{m}a_{\mathrm{ik}}\left(\mu \right)f_{k}\right)-{\mathrm{SC}}_{i}-\sum_{k\in j=1}^{m}a_{\mathrm{ik}}\left(\mu \right)f_{k}-\mathrm{Ln}\left(g_{i}\right)\right]$$

Now, we obtain * s*uch that this vector maximizes the LML function. The Vector satisfies Equation 4 which is a necessary condition to maximize the LML function:

Equation 4 $$\frac{\partial L}{{\partial f}_{j}}=g_{i}\frac{\beta_{\mathrm{ij}}+a_{\mathrm{ij}}\left(\mu \right)}{{\mathrm{SC}}_{i}+\sum_{k\in j=1}^{m}a_{\mathrm{ik}}\left(\mu \right)f_{k}}-\beta_{\mathrm{ij}}-a_{\mathrm{ij}}\left(\mu \right)=0$$

j=1,2,…, m

Applying in equation 4, we can obtain the following MLEM iterative formula for reconstruction of the SPECT images:

Equation 5 $${f_{j}}^{\left[K+1\right]}=\frac{{f_{j}}^{\left[K\right]}}{\sum_{i=1}^{n}a_{\mathrm{ij}}\left(\mu \right)}\sum_{i=1}^{n}(g_{i}\frac{\beta_{\mathrm{ij}}+a_{\mathrm{ij}}\left(\mu \right)}{{\mathrm{SC}}_{i}+\sum_{k\in j=1}^{m}a_{\mathrm{ik}}\left(\mu \right){f_{k}}^{\left[K\right]}}-\beta_{\mathrm{ij}})$$

 j=1,2,…,mK=0, 1, 2,…  j=1,2,…,m K=0,1,2,…

Where * , j=1,2,...,m* is the initial guess for the activity of pixels of SPECT image. It should be note that if *SC*_i_*=0* then *=0* and the proposed MLEM iterative formula is converted to the MLEM formula.

Equation 6 $${f_{j}}^{\left[K+1\right]}=\frac{{f_{j}}^{\left[K\right]}}{\sum_{i=1}^{n}a_{\mathrm{ij}}\left(\mu \right)}\sum_{i=1}^{n}(g_{i}\frac{a_{\mathrm{ij}}\left(\mu \right)}{\sum_{k\in j=1}^{m}a_{\mathrm{ik}}\left(\mu \right){f_{k}}^{\left[K\right]}})$$

**Figure 1:**
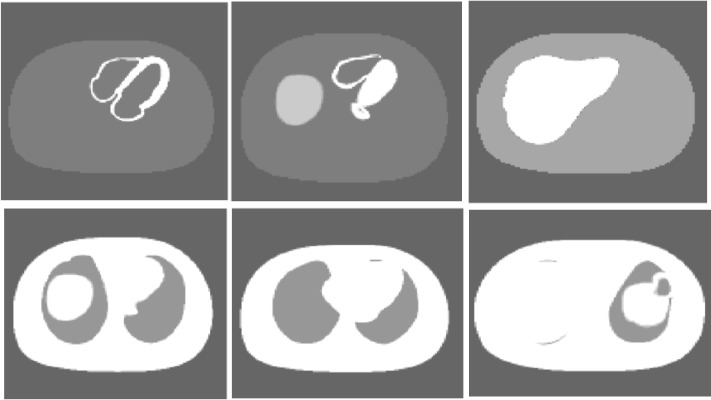
Three trans-axial slices of NCAT phantom; Activity distribution (top row) and their corresponding attenuation map (bottom row)

***Phantom studies***

A digital NCAT and NEMA image quality phantoms were used in this work.

*A) Digital phantom*

The NURBS-based cardiac-torso (NCAT) phantom was used to assess the performance of our suggested formula in realistic conditions (12). It can produces attenuation and an activity distribution map of the organs with user defined data (Figure 1). In activity distribution map, ^99m^Tc activity ratio was 100, 100, 40, 22, 6 and 6 in myocardium, gallbladder, liver, spleen, lung and background respectively (13). The attenuation map was generated for photon energy of 140 keV. The phantom dimension was 40×40×20 cm^3^ that was digitized into 128×128×64 voxels. The Monte Carlo simulation program was based on a published paper by Kalantari *et al* (11). 

*B) Experimental phantom: image quality phantom*

**Figure 2:**
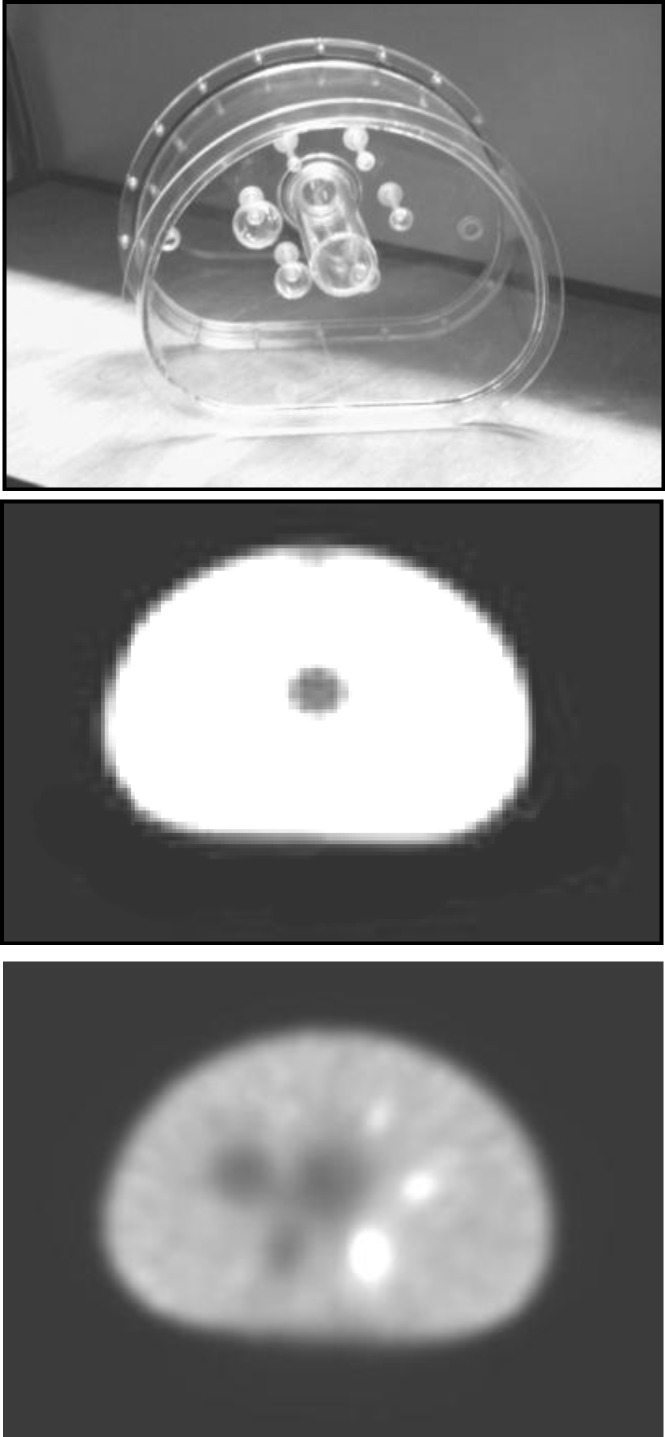
An image quality phantom (up); attenuation map (middle) and its activity distribution (down

A GE‘s Infineon Hawkeye SPECT/CT scanner was used to acquire row projection data from the NEMA image quality phantom (Figure 2). The phantom has a roughly elliptical shape and contains six fillable spheres of varying sizes with inner diameters of 10, 13, 17, 22, 28 and 37 mm (14). The background and 4 hot spheres of the phantom (inner diameters of 10, 13, 17, 22 mm) were filled with ^99m^Tc, with an activity/concentration ratio of 8:1, the cold spheres activity was zero. So, the activity map of this phantom contains uniform background, 4 hot spheres, 2 cold spheres, and an absorber in the center. Projection data of the phantom were measured with 64×64 pixels, from 0-360° with 6 degree increments. 

***Attenuation and scatter correction***

The CT based attenuation correction was performed in this work for the NEMA image quality phantom study (15-17), using bilinear method energy mapping (18-20). 

Correction for attenuation in NCAT phantom was based on the attenuation map, generated during simulation.

Scatter distribution of a specified pixel element j from neighbor pixels, along a particular ray-of-view, bin i, for any projection was calculated by Klein-Nishina formula (21). 

***Image reconstruction methods***

 In this work, we applied five different image reconstruction algorithms based on the Maximum Likelihood Expectation Maximization (MLEM) algorithm:

- Reconstruction of row projections data via MLEM without any correction. They are named "Reference" when reconstructing projections consist of only primary photons and "Un_Cor" when reconstructing projections consist of primary and scatter photons.
- Reconstruction with attenuation correction of row data (Equation 6), using specific attenuation map and producing $a_{\mathrm{ij}}\left(\mu \right)$ as attenuated system matrix elements. They are named "Att_Cor".
- Reconstruction with attenuation and scatter correction of row data via conventional formula (Equation 7) and named "Conventional" (22).

Equation 7 $${f_{j}}^{\left[K+1\right]}=\frac{{f_{j}}^{\left[K\right]}}{\sum_{i=1}^{n}a_{\mathrm{ij}}}\sum_{i=1}^{n}(a_{\mathrm{ij}}\frac{g_{i}}{\sum_{k\in j=1}^{m}a_{\mathrm{ik}}{f_{k}}^{\left[K\right]}+{\mathrm{SC}}_{i}}),$$

i=1,2,…, n j=1,2,…, m           

Where *SC*_i_ is the estimated scatter projection. In this study, *SC*_j_ was updated during the iterations.

- Reconstruction with attenuation and scatter correction of row data via our proposed MLEM iterative formula with inter-slice scatter estimation (New1, Equation 5).
- Reconstruction with attenuation and scatter correction of row data via our proposed MLEM iterative formula using 3 slices scatter estimation (New3, Equation 5).

For both, the NCAT simulation and the image quality phantom measurement a 360° SPECT acquisition with 60 different projection angles were used for reconstruction.

***Evaluation parameters***

Six different parameters were applied to compare the different images in this work. They are profile agreement, contrast, mean square error (MSE), signal-to-noise ratio (SNR), contrast-to-noise ratio (CNR) and computational time used for image reconstruction.

*Profile agreement*

Horizontal profiles will show the activity distribution and are used to evaluate the agreement between the proposed MLEM method-corrected slice images (NEW1 and NEW3) and the reference image in NCAT phantom.

Furthermore, horizontal profiles are used to evaluate the agreement between the corrected slice images of proposed MLEM method (NEW1 and NEW3) and conventional MLEM formula in both NCAT and image quality phantoms.

*Contrast*

The contrast was calculated as described by Wieczorek (9) with the following formula:

Equation 8 $$\mathrm{Ctr}=\frac{\frac{\mathrm{N2}}{\mathrm{n2}}}{\frac{\mathrm{N1}}{\mathrm{n1}}}-1$$

Where *N*_2_ and *N*_1_ are the sum of pixel values in the particular hot and background region, respectively, and *n*_i_ is the number of pixel elements.

**Figure 3:**
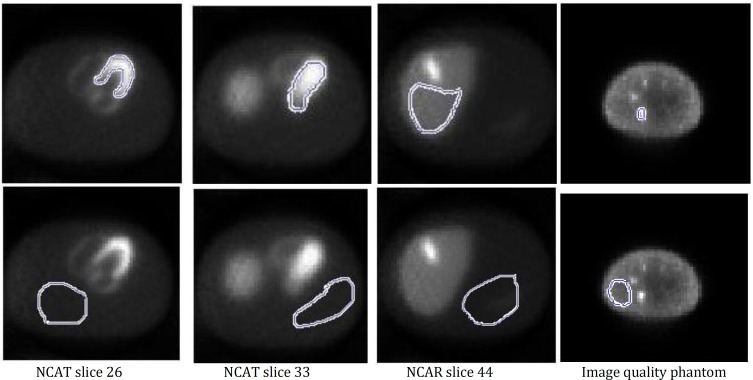
Region of interest in different slices, the regions of interest in the first row were used to calculate the signal and the regions of interest in the second row were used to calculate noise

*Mean squared error (MSE)*

To evaluate the similarity between each reconstructed image (P) and reference image (T), MSE was defined as the average of the square differences(23).

Equation 9 $$\mathrm{MSE}=\frac{1}{n_{x}n_{y}}\sum_{x=1}^{n_{x}}\sum_{y=1}^{n_{y}}{\left((P\left(x,y\right)-T\left(x,y\right)\right)}^{2}$$

Where n_x _and n_y _are the number of image pixels in each row and column, respectively.

*Signal-to-noise ratio (SNR)*

Here, the SNR is defined as the ratio of mean signal to standard deviation of the background. Different region of interest (ROI) were selected along the heart wall, left part of the heart, liver and the biggest hot sphere in 26th, 33th, 44th slices of NCAT phantom and in the image quality phantom, respectively. Another ROI in the background was selected for all slices. These ROIs were used for different quantitative measurements of this study. 

*Contrast-to-Noise Ratio*

The contrast-to-noise ratio (CNR) is defined as follows (24).

Equation 10 $$\mathrm{CNR}=\frac{\mathrm{Contrast Recovery Coe}ﬃ\mathrm{cient}}{\mathrm{Coefficient Of Variation}}$$

Where $\mathrm{Contrast Recovery Coefficient}=\frac{\frac{m_{l}-m_{b}}{m_{b}}}{C-1}$

and *m*_l_ and *m*_b_ are the mean lesion (hot) and background activity, *C* is the real contrast in the phantom and coefficient of variation is defined as the ratio of the standard deviation to the mean.

*Computational time*

The reconstruction time per iteration for different methods was determined using the MATLAB software package.

## Results

*Activity distribution and profile agreement*

**Figure 4:**
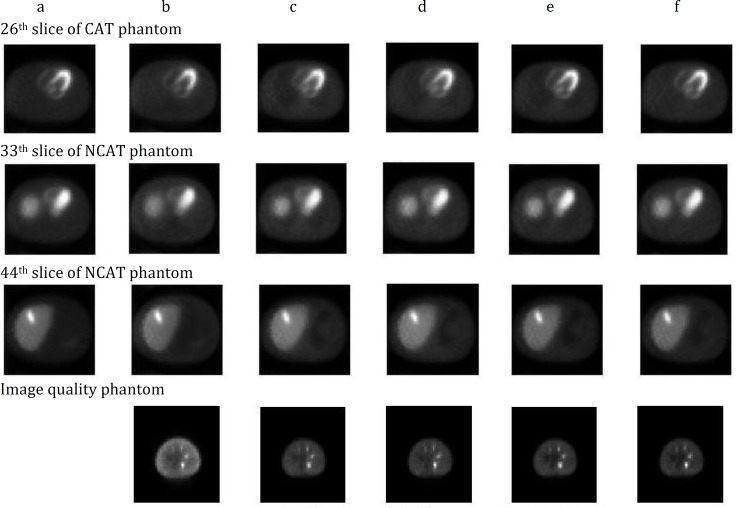
Images resulted from different reconstruction methods: a)Reference, b)Un_Cor, c)Att_Cor, d) Conventional, e) New1, f) New3

Figure 4, from top to bottom, shows tomographic images of the 26th, 33th and 44th slice of NCAT and one slice of image quality phantom, respectively.

Reconstructed images resulted from different MLEM reconstruction methods have been shown in different columns of Figure 4. The images from left to right are reference (for simulated slices only), Un_Cor, Att_Cor, Conventional, New1, and New3.

**Figure 5:**
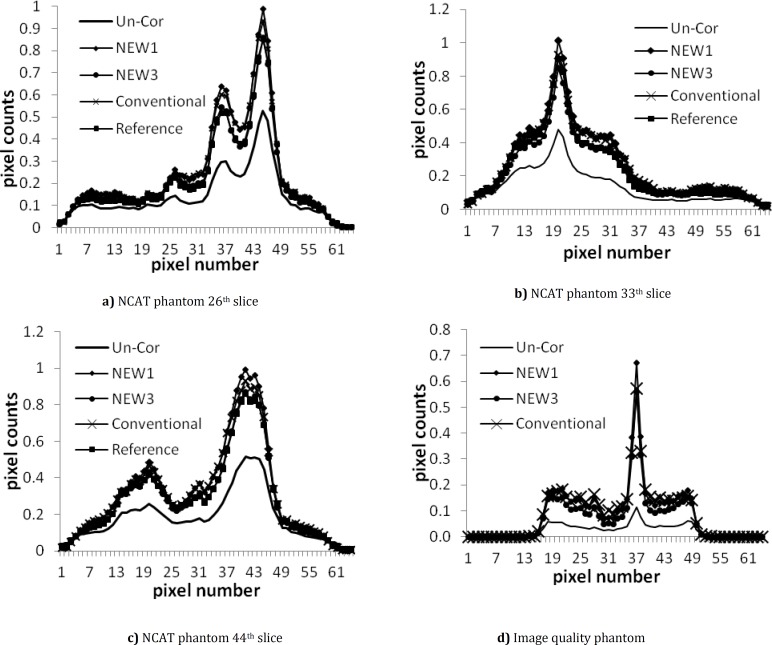
Horizontal profile of 26^th^, 33^th^ and 44^th^ slices of NCAT and image quality phantoms

 The horizontal profiles passing through 26^th^ row of each slice image (Figure 5) show a good agreement between profiles of the corrected images resulted from the new MLEM formula and the results from conventional MLEM. The misplacement of events has decreased significantly by scatter correction and the contrast enhanced according to Table 1. Figure 5 and Table 1 show that the image reconstructed without scatter compensation had more over-estimation of counts and loss of contrast due to the presence of scatter in comparison with Un_Cor and Att_Cor. 

*Mean squared error (MSE)*

**Figure 6:**
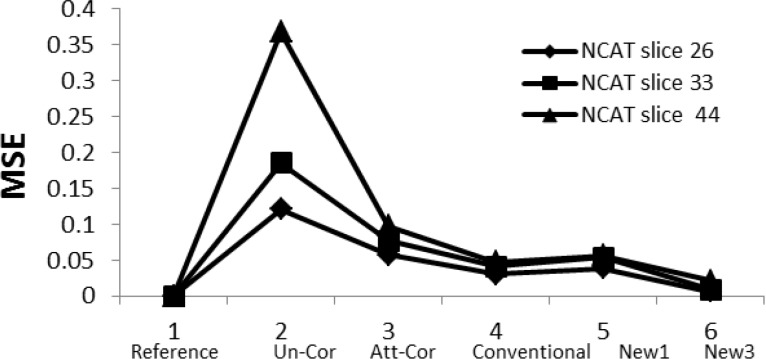
Mean square error of the images against different reconstructed methods

 MSE between the reference image and other reconstructed images shows the same behavior in all slices (Figure 6). 

The lower the value of the MSE the better is the similarity of the reconstructed image with the reference image. According to this, the differences between the reference and Un_Cor images are the greatest and also the differences between the reference and the scatter and attenuated corrected images are the smallest. 

**Table 1:** Contrast of images resulted from different reconstruction methods

Contrast	Reference	No_cor	Att_cor	Conventional	New1 slice	New3 slices
Image quality phantom	----------	2.8	4.0	3.9	5.1	5.5
NCAT 26	6.8	5.6	6.1	6.0	6.3	6.2
NCAT 33	6.1	4.9	5.6	5.5	5.9	6.0
NCAT 44	8.1	6.7	6.3	6.3	6.8	6.6
						

**Figure 7:**
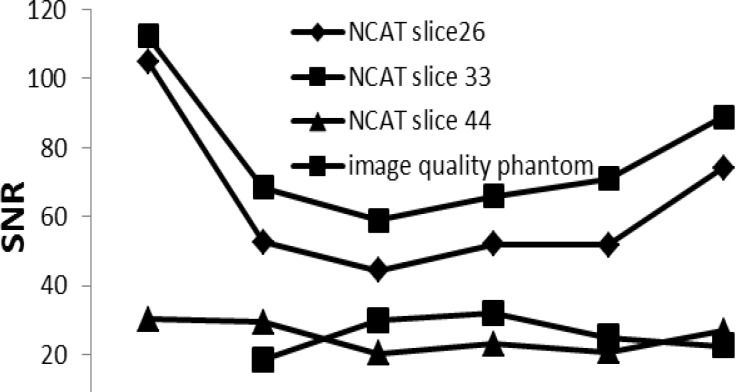
Signal-to-noise ratio of the images against different reconstructed methods

**Figure 8:**
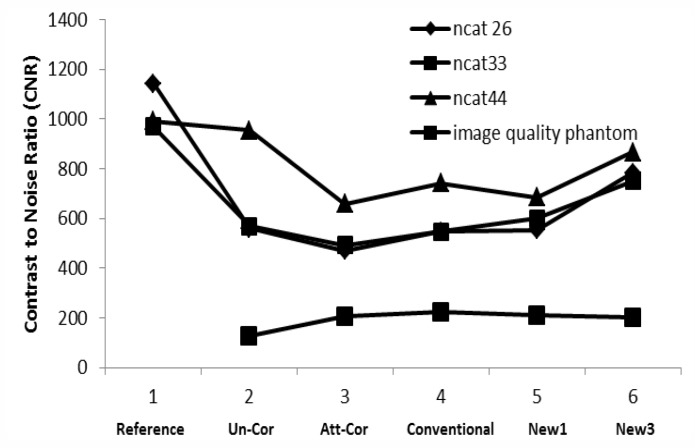
Contrast-to-noise ratio of the images against different reconstructed methods

*Signal_to_noise (SNR) and contrast-to-noise ratio (CNR)*

Signal_to_noise ratio of NCAT phantom based on simulated data in Figure 7 and contrast-to- noise ratio of NCAT and image quality phantom in Figure 8 show that our new approaches (New1 and New3) have comparable accuracy with the conventional formula.

CNR of Un_Cor, Att_Cor, Conventional, New 1 and New 3 are 127.00, 206.65, 222.28, 208.92 and 201.82 respectively.

*Computation time*

Computation time required for reconstruction of one slice image (64×64 pixels) from a set of 64 projections in the last iteration is 0.029, 0.118 and 0.123 s for Conventional, New1 and New3 respectively. This means that using new formula, the computation time increases by a factor of 4, but it is still in a reasonable time for clinical purpose.

## Discussion

In this study, we introduce a novel reconstruction formula implementing attenuation and scatter correction of row data with inter-slice scatter estimation (namely New 1 and New 3, Equation 5).

The new algorithm was tested by a simulation of the NCAT phantom and the SPECT acquisition of the NEMA image quality phantom.

As follows, six different parameters were measured in the final images to quantify and compare the results of the different reconstruction algorithms.

- Profile agreement
- Contrast
- Mean Square Error (MSE)
- Signal-to-Noise ratio (SNR)
- Contrast-to-Noise ratio (CNR)
- Computational time necessary for image reconstruction

The applicability of the presented algorithm is shown by well agreement of horizontal profiles (Figure 5).

The scatter and attenuation compensated images show a better contrast (with the mean of 25% increase) than the uncorrected images allowing a better delineation of the lesions in the scatter and attenuation-compensated images. This is in well agreement with the trials demonstrated that scatter and attenuation can increase the contrast in SPECT studies (11). Also, in comparison with the reference slice, the images resulted from the presented new method show a slightly better contrast. 

The MSE values were reduced by 52.9% to 94.3% in the corrected images as compared with the reference images (Figure 6) which is in agreement with other studies (11, 25). MSE was reduced from 0.4 in the Un_Cor to 0.09, 0.04, 0.05 and 0.02 in the Att_Cor, Conventional, New1 and New3, respectively as compared with the reference images (in the slice 44). The same behavior is observed in other slices. The obtained results also point to the importance of scatter and attenuation correction together during reconstruction of images, compared with attenuation correction only without scatter correction. Signal-to-noise study, based on the simulated data, shows that all the scatter and attenuation corrected images have up to 67.1% higher SNR in comparison with the Att_Cor slice images (Figure 7). 

Contrast_to_noise (CNR) study also shows that all the scatter and attenuation corrected images have up to 52.5% higher CNR in comparison with Att_Cor images (Figure 8). This is in agreement with several trials demonstrated that scatter correction (26, 27), attenuation correction (11) and both correction applied together (28, 29) can significantly increase the CNR. Due to more accurate scatter estimation in New3, the resulting images show the best CNR among other corrected images. 

## Conclusion

The proposed formula for incorporating scatter and attenuation during MLEM, enables to remove scatter and compensate attenuation as a necessary step for quantitative SPECT images. The new mathematical method presented in this study increases the contrast, SNR, and CNR of the images and decreases the MSE in comparison with Un_Cor and Att_Cor images.
